# Chicago Pathological Society

**Published:** 1884-04

**Authors:** J. H. Tebbetts

**Affiliations:** Secretary


### Society Reports.

### ARTICLE IX.

CHICAGO PATHOLOGICAL SOCIETY.

Regular meeting, October 8, 1883.

The Society was called to order by the President, Dr. Angear,

The minutes of the last two meetings were read and approved. Dr. Fannie Dickinson was elected to membership unanimously.

Dr. F. C. Knight was proposed for membership by Dr. G. F. Lydston and the Secretary.

The Society then listened to the reading of a very interesting paper by Dr. H. M. Lyman, on Apoplexy, in illustration of which the author gave a detailed account of numerous interesting cases. In diagnosis one needs to exercise careful judgment: alcoholic intoxication and an apoplectic seizure may coexist. Hysterical women have fallen in an attack of unconsciousness, with resulting hemiplegia, which in a short time may be transferred to the other side of the body.

Apoplexy may be simulated by uræmic poisoning. In apoplexy the temperature first falls, then rises. In treating the worst cases nothing is of avail; in those milder, we may employ leeches, wet cups, general bleeding only when the right heart is involved.

Attention should be directed to the state of the bladder, and the bowels be kept open.

For medication, give moderate doses chlorate of potassium, and watch its action on the kidneys; also tinct. aconite root. In the stage of convalescence, dietetic general treatment, electricity, and massage.

The paper being before the Society for discussion, the President, Dr. Angear, marked his interest in the paper and its subject matter. To the profession the diagnosis of apoplexy is all-important, and in some cases, when so diagnosticated, apoplexy had not occurred.

He had not a large experience, but a case seen years ago would bear relating, in which a lady suffering from headache, and reflex pains in the left shoulder and arm, had been treated by her physician for uterine disease, and was suddenly seized with apoplexy and death, the post-mortem revealing this condition. The disease of the brain is partly revealed by these obscure symptoms, disturbances of the mucous membrane, as well as interference in the nutrition of the skin, on account of which bed-sores form quickly. Fatal syncope is often diagnosticated as apoplexy. The diagnosis should be carefully studied. In another case, when hastily summoned, he found a man speechless and hemiplegic, the eye natural and senses unclouded, which condition was quickly relieved by an emetic, and the throwing up of a large amount of sauerkraut, beer, etc.; had death occurred the diagnosis would probably have been made of apoplexy, and that of the nervous variety may have really existed.

A member of the County Hospital staff present then spoke of a case of medico-legal interest seen lately. The patient was in coma, hemiplegic, with all symptoms of apoplexy, and had been picked up in this condition on the street. No signs of foul play were noticed. Remained thus forty-eight hours, then died. Post-mortem examination revealed a large blood clot upon left lobe of brain, and a fracture of the skull through the left parietal to the temporal bone. No bruises were visible on the scalp. The cause of the fracture may have been a blow from a sand-bag.

Dr. C. J. Lewis thought Dr. Lyman had given all the points to bear in mind in diagnosis, and briefly related a case of a man whose skull was fractured by a fall in leaping from a window; the symptoms were of apoplexy, hemiplegia, etc. In trephining there was found extensive vascular rupture producing great pressure, although there was but slight pressure from the bone.

Dr. Tebbetts briefly spoke of two recent cases; after which the society proceeded to consideration of miscellaneous business.

The President remarked that as there might be some complaint made during the winter on account of the present location of the society being insufficiently central, and as the present place of meeting had been secured experimentally, he suggested the consideration of the matter at present meeting.

Dr. Lyman moved that the officers of the society be appointed a committee to look up a suitable hall or other convenient place for the society, and report at the next meeting. Carried.

Dr. Lyman's name was, by motion, added to the above committee.

Among those present were Drs. Angear, Beecher, Dobbin, Harper, Lewis, Lyman, Treat, Tebbetts, and several visitors.

The society, on motion, adjourned.

J. H. TEBBETTS, Secretary.

---

CHICAGO PATHOLOGICAL SOCIETY.

